# A novel approach to determine two optimal cut-points of a continuous predictor with a U-shaped relationship to hazard ratio in survival data: simulation and application

**DOI:** 10.1186/s12874-019-0738-4

**Published:** 2019-05-09

**Authors:** Yimin Chen, Jialing Huang, Xianying He, Yongxiang Gao, Gehendra Mahara, Zhuochen Lin, Jinxin Zhang

**Affiliations:** 1 Department of Medical Statistics and Epidemiology, School of Public Health, Sun Yat-sen University, Guangzhou, 510080 China; 2 National Engineering Laboratory for Internet Medical Systems and Applications, the First Affiliated Hospital of Zhengzhou University, Zhengzhou, 450052 Henan China

**Keywords:** Optimal cut-points, Discretization, Categorize, U shape, Cox regression model, Survival analysis

## Abstract

**Background:**

In clinical and epidemiological researches, continuous predictors are often discretized into categorical variables for classification of patients. When the relationship between a continuous predictor and log relative hazards is U-shaped in survival data, there is a lack of a satisfying solution to find optimal cut-points to discretize the continuous predictor. In this study, we propose a novel approach named optimal equal-HR method to discretize a continuous variable that has a U-shaped relationship with log relative hazards in survival data.

**Methods:**

The main idea of the optimal equal-HR method is to find two optimal cut-points that have equal log relative hazard values and result in Cox models with minimum *AIC* value. An R package ‘CutpointsOEHR’ has been developed for easy implementation of the optimal equal-HR method. A Monte Carlo simulation study was carried out to investigate the performance of the optimal equal-HR method. In the simulation process, different censoring proportions, baseline hazard functions and asymmetry levels of U-shaped relationships were chosen. To compare the optimal equal-HR method with other common approaches, the predictive performance of Cox models with variables discretized by different cut-points was assessed.

**Results:**

Simulation results showed that in asymmetric U-shape scenarios the optimal equal-HR method had better performance than the median split method, the upper and lower quantiles method, and the minimum *p*-value method regarding discrimination ability and overall performance of Cox models. The optimal equal-HR method was applied to a real dataset of small cell lung cancer. The real data example demonstrated that the optimal equal-HR method could provide clinical meaningful cut-points and had good predictive performance in Cox models.

**Conclusions:**

In general, the optimal equal-HR method is recommended to discretize a continuous predictor with right-censored outcomes if the predictor has an asymmetric U-shaped relationship with log relative hazards based on Cox regression models.

**Electronic supplementary material:**

The online version of this article (10.1186/s12874-019-0738-4) contains supplementary material, which is available to authorized users.

## Background

In survival analysis, Cox regression models [1], which are the most popular model in this field, are frequently used to investigate the effects of explanatory variables on right-censored survival outcomes. The explanatory variables may be continuous, such as age or weight, or they may be discrete variables, such as gender or treatment factors. When continuous explanatory variables have nonlinear effects on outcomes, it is of interest to investigate U-shaped relationships [2–5] between continuous explanatory variables and health-related outcomes in many researches. Although the U-shaped effects of continuous variables can be modeled in Cox models with flexible smoothing techniques [6–8], such as penalized splines and restricted cubic splines, many clinical and epidemiological researchers would rather discretize continuous explanatory variables [9, 10] to reflect high-risk and low-risk values of the independent variables and compare the risks of developing survival outcomes (i.e. deaths or relapses) between different groups of patients. Moreover, optimal cut-points could help identify thresholds of important predictors, which could be used to provide classification schemes of the patients and assist in making clinical treatment decisions. In practice, it is sensible to use standard clinical reference values as cut-points to discretize continuous predictors. But when it comes to lack of standard reference ranges for newly discovered risk factors or the reference ranges can’t be applied to the population with different characteristics, how to find the scientific and reasonable cut-points to categorize continuous independent variables has been an important issue to be addressed [11–13].

There are two widely adopted approaches to discretize continuous independent variables in survival analysis. One is the data-oriented cut-points approach [14, 15], which uses the median value, quartiles or other percentile values based on the distribution of continuous variables as cut-points. Owing to its simplicity and easiness of implementation, median value and upper and lower quantiles (noted as Q1Q3) have been widely used in many studies as cut-points. However, this approach provides arbitrary cut-points regardless of the relationships with survival outcomes and might lead to wrong estimates of the true effects. Another approach named maximum statistic approach or minimum *p*-value approach was first developed by Miller and Siegmund [16] to dichotomize continuous predictors with binary outcomes. The minimum *p*-value approach selects a cut-point with maximum *χ*^2^ statistic as the optimal cut-point when the outcomes are binary. When it is extended to survival outcomes, the optimal cut-point is the one that results in a minimum *p*-value of log-rank tests [17]. In the simulation studies of the minimum *p*-value approach, it is usually assumed that there is a single theoretical threshold of continuous variables, which means relationships between independent variables and survival outcomes are stepwise functional relations. In practice, independent variables and survival outcomes generally have smooth relationships instead of biologically implausible stepwise functional relationships. In addition, U-shaped relationships between continuous variables and outcomes are commonly seen in the clinical and epidemiological studies [2–5] but little considered in the study of the discretization methods. In the case of body mass index (BMI), a too low and a high BMI value both cause harmful effects on overall health [3, 18]. When a prognostic variable has a U-shaped relationship with outcomes, the effect of the prognostic variable may be underestimated using high and low-risk groups divided by a single cut-point.

**Fig. 1:**
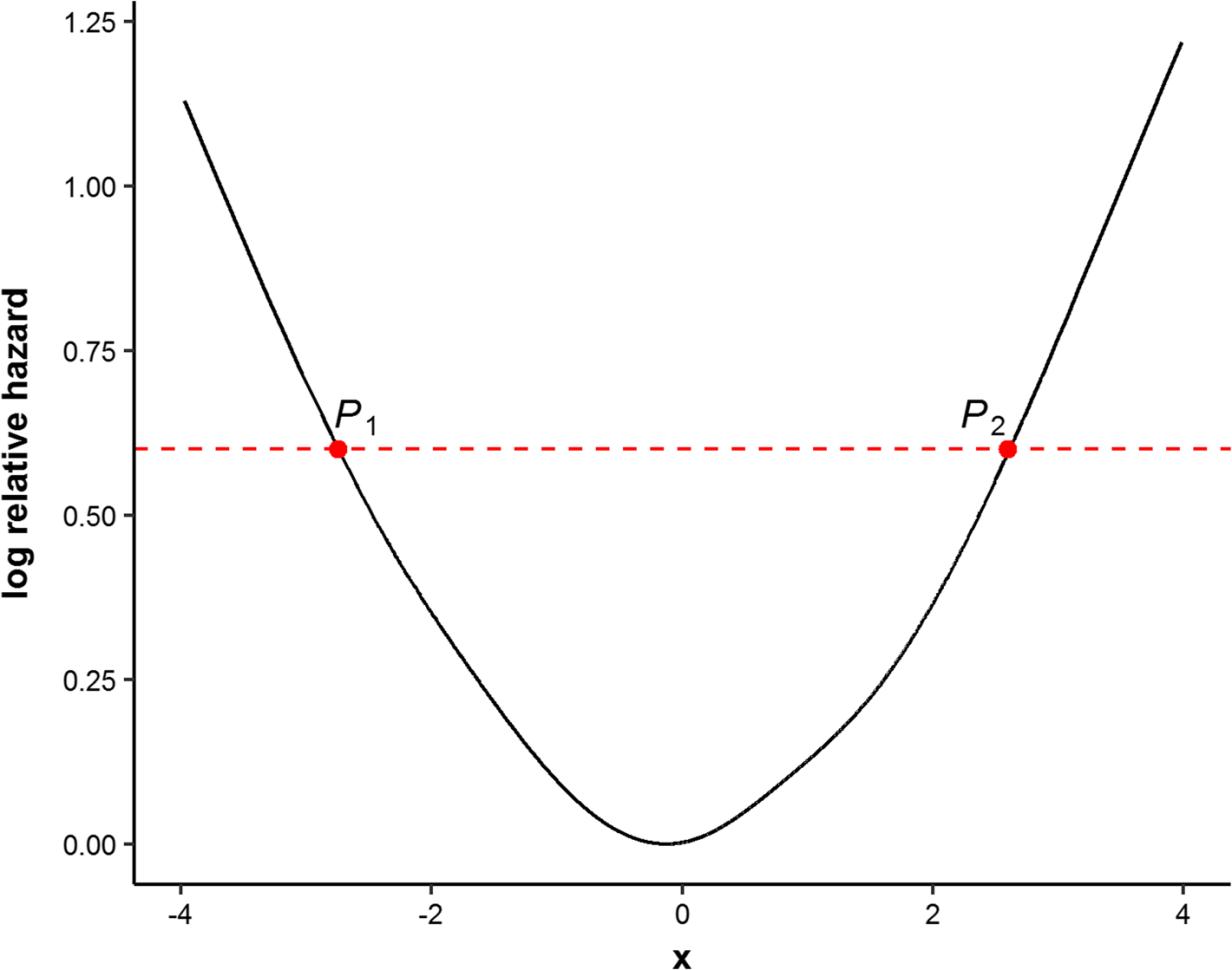
Schematic diagram of the optimal equal-HR method. The solid black line presents the U-shaped relationship between the continuous x and log relative hazard. The red dashed line is parallel to the x-axis, which means *P*_1_ and *P*_2_ have equal log relative hazard values. The optimal equal-HR method searches pairs of cut-points with equal log relative hazard values as candidate cut-points, such as (*P*_1_, *P*_2_)

To overcome the shortcomings of the common discretization methods in survival data and meet the needs of finding optimal cut-points for a continuous predictor that has a U-shaped relationship with survival outcomes, we propose a new approach named optimal equal-HR method to estimate two optimal cut-points that have approximately equal log hazard values based on Cox regression models. The main idea of the optimal equal-HR method is derived from the clinical need of classifying patients into high-risk and low-risk groups according to a categorized prognostic variable. In clinical practice, the classification of high-risk or low-risk patients is based on their risks of developing unfavored survival outcomes, such as deaths or relapses. And the larger log relative hazards correspond to the higher risks of developing the observed survival outcomes. Despite the reference ranges, common methods for classification in practical epidemiology studies, such as the Q1Q3 method, are not accurate to define such low-risk or high-risk groups when there exists a U-shaped relationship. It is because the individuals with the same risks (the same log relative hazards) could be divided into opposite risk groups by the Q1Q3 method. During the procedures of finding optimal cut-points, it is important to consider the effects of log relative hazards. Hence, we use log relative hazards to find two optimal cut-points (*P*_1_, *P*_2_) of the prognostic variable *X* (as shown in Fig. 1). The patients with *X* smaller than *P*_1_ or larger than *P*_2_ are classified into two high-risk groups (or two low-risk groups in an inverse U-shaped relationship) respectively. By doing this, the two high-risk groups are defined according to the same threshold of log relative hazards, which makes the classification more reasonable in clinical explanations. Our method can provide more accurate optimal cut-points and avoid the individuals with the same risks (the same log relative hazards) being divided into opposite risk groups. An R package named ‘CutpointsOEHR’ has been originally developed to help investigators easily implement the optimal equal-HR method.

The rest of this paper is organized as follows. The details of the optimal equal-HR method are presented in next Section ‘Methods’. The performance of the optimal equal-HR method is compared with other commonly used discretization methods regarding discrimination power and overall performance via a simulation study. We present the simulation settings in Section ‘The Simulation Study’. The results of the simulation study and the application of the optimal equal-HR method on a real dataset of small cell lung cancer are presented in Section ‘Results’. Finally, there are discussion and conclusions.

## Methods

The optimal equal-HR method is based on Cox proportional hazards regression models [1] which have the following structure:

1 $$ h(t)={h}_0(t)\mathit{\exp}\left({\beta}^{\prime }X\right) $$

where *h*(*t*) denotes the hazard function, *h*_0_(*t*) denotes the baseline hazard function, *t* is the observed survival times, *X* is a vector of covariates, and *β* is a vector of estimated regression coefficients. The relative hazard *λ* can be calculated as the above equation divided by *h*_0_(*t*) in both side:

2 $$ \lambda =\frac{h(t)}{h_0(t)}=\mathit{\exp}\left({\beta}^{\prime }X\right) $$

The optimal equal-HR method uses log(*λ*) values to search for optimal cut-points. Hazard ratios (HR) could be easily calculated from the relative hazards *λ* when investigators choose a reference value of an independent variable and control other variables at average levels. And the relationship between HR and a continuous variable is identical to that between *λ* and the continuous variable. Therefore, the method of finding the optimal cut-points with approximate equal log(*λ*) values is named as optimal equal-HR method. The procedure of the optimal equal-HR method contains two main steps described as follows.

### Graphical diagnostic plot

The optimal equal-HR method proposed in this study aims to solve the problem of discretizing a continuous variable that has a U-shaped relationship with log(*λ*) in the Cox model. Therefore, the first step of adopting the optimal equal-HR method is to determine the relationship between a continuous covariate and log(*λ*) and plot the curve. Previous researches have already proposed several methods for estimating nonlinear relationships, including the multiple *β* method [19], martingale residual based method [20], spline methods [21], etc. The performance of the multiple *β* method for Cox models is unstable and largely depends on the number of selected groups because a Cox model is a semi-parametric model and its likelihood is based on the order of events rather than their distributions. The martingale residual based method, which uses martingale residuals from Cox models to test the log-linearity, cannot plot the relationship between a continuous covariate and log(*λ*). Due to the limitations of the above two methods, this study used Cox regression models with penalized *B*-splines (*P*-splines) [7, 22], which balances goodness of fit and variance, to curve the relationship and determine whether the non-linear term is statistically significant. The smoothing parameter of the *P*-splines of degree 3 with 22 evenly spaced knots is automatically chosen by minimizing *AIC*, which is achieved through the R-function ‘pspline’ in the ‘suevival’ package under the univariate situation. If there are two or above covariates with nonlinear effects, the R-function ‘dfmacox’ in the ‘smoothHR’ package [23] could be used to obtain the optimal smoothing parameter. Then the estimated log(*λ*) values are plotted against the continuous variable to give an insight into the biological nature of the continuous variable.

### Find two optimal cut-points

If the plotted curve suggests of a U-shaped relationship (such as Fig. 1), two optimal cut-points of the continuous variable are searched based on the relationship curve of the continuous variable and log(*λ*). Specific steps of the optimal equal-HR method are as follows:

1. Calculate the percentiles of the estimated log(*λ*) values, denoted as *Q*_*k*_, *k* = 1, 2, ⋯, 100. For each percentile value between the 5th and the 95th percentile of the estimated log(*λ*), draw a straight line parallel to the x-axis, y = *Q*_*k*_, *k* = 5, 6, … , 95. The line crosses the fitted U-shaped curve with two intersections (illustrated in Fig. 1). The two observations ${P}_{1k}\left({X}_{1k},\mathit{\log}\left(\widehat{\lambda}|X={X}_{1k}\right)\right),{P}_{2k}\left({X}_{2k},\mathit{\log}\left(\widehat{\lambda}|X={X}_{2k}\right)\right),{X}_{1k}<{X}_{2k}$, which are closest to the two intersections respectively, are found as a pair of candidate cut-points with a constraint that $\left|\log \left(\widehat{\lambda}|X={X}_{1k}\right)-\log \left(\widehat{\lambda}|X={X}_{2k}\right)\right|\le 0.01$. If the constraint is violated, the linear interpolate method is used to construct new data points as candidate cut-points, which ensures candidate cut-points have equal log(*λ*) values.
2. The continuous predictor *X* is discretized into a categorical covariate *X*^′^ with low range (*X* < *X*_1*k*_), median range (*X*_1*k*_ < *X* < *X*_2*k*_), and high range (*X* > *X*_2*k*_) according to each pair of candidate cut-points.
3. Then the categorical covariate *X*^′^ (reference level is the median range) is fitted in a Cox model and the concomitant Akaike Information Criterion (*AIC*) value is calculated. The pair of cut-points that minimizes *AIC* values is defined as optimal cut-points. Moreover, choosing cut-points by the Bayesian information criterion (*BIC*) has the same results as *AIC* (Additional file 1: Tables S1, S2 and S3).

### Implementation in R

The optimal equal-HR method was implemented in the language R (version 3.3.3). The freely available R package ‘survival’ was used to fit Cox models with *P*-splines. The R package ‘pec’ was employed for computing the Integrated Brier Score (*IBS*). The R package ‘maxstat’ was used to implement the minimum *p*-value method with log-rank statistics. And an R package named ‘CutpointsOEHR’ was developed for the optimal equal-HR method. This package could be installed in R by coding *devtools::install_github(“yimi-chen/CutpointsOEHR”).* All tests were two-sided and considered statistically significant at *P* < 0.05.

## The simulation study

A Monte Carlo simulation study was used to evaluate the performance of the optimal equal-HR method and other discretization methods including the median split (Median), the upper and lower quartiles values (Q1Q3), and the minimum log-rank test *p*-value method (min*P*). We generated right-censored survival data with known U-shaped exposure-response relationships. To investigate the performance of these methods, the predictive performance of Cox models fitted with different discretized variables was assessed.

### Design of the simulation study

The survival times *T*_0_ were generated from the Weibull distribution using the method from Bender’s research [24] as

4 $$ {T}_0={\left(-\frac{\log (U)}{\lambda\ \exp \left(s(x)\right)}\right)}^{1/v} $$

where *U* followed a uniform distribution on the interval from 0 to 1, abbreviated as *U*~U(0, 1), *λ* was the scale parameter of Weibull distribution, *v* was the shape parameter of Weibull distribution, *x* was a continuous covariate from a standard normal distribution, and *s*(*x*) was the given function of interest. To simulate U-shaped relationships between *x* and log(*λ*), the form of *s*(*x*) was set to be

5 $$ s(x)=\left\{\begin{array}{c}{k}_1x,x\le a\\ {}{k}_2x,\kern0.5em x>a\end{array}\right. $$

where parameters *k*_1_, *k*_2_ and *a* were used to control the symmetric and asymmetric U-shaped relationships. When -*k*_1_ was equal to *k*_2_, the relationship was almost symmetric. For each subject, censoring time *C* was simulated by the uniform distribution with [0, *r*]. The final observed survival time was *T* = min(*T*_0_, *C*), and *d* was a censoring indicator of whether the event happened or not in the observed time *T* (*d* = 1 if *T*_0_ ≤ *C*, else *d* = 0). The parameter *r* was used to control the censoring proportion *P*_c_.

**Fig. 2:**
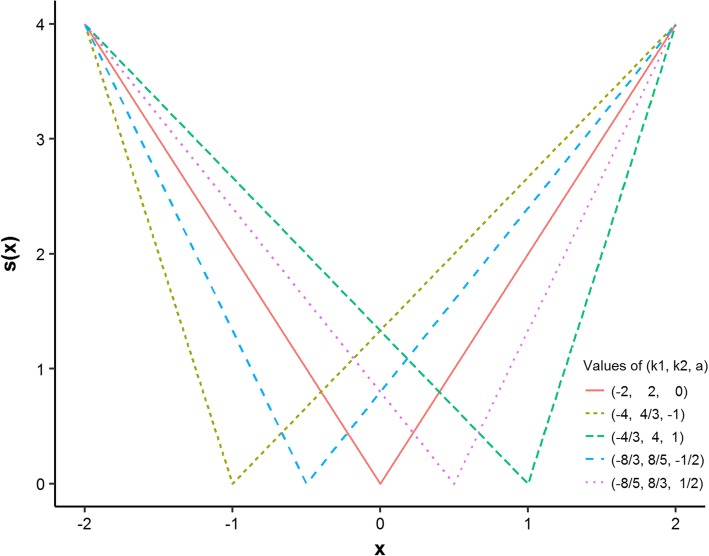
Different forms of s(x) in the simulation. Five forms of s(x) are used to simulate symmetrical and asymmetrical U-shaped relationships between a continuous variable and log relative hazards. Parameter *k*_1_ is the slope of the left line in a U-shaped or V-shaped curve, parameter *k*_2_ is the slope of the right line and parameter *a* is the value of the turning point in the x-axis

One hundred independent datasets were simulated with *n* = 500 subjects per dataset for various combinations of parameters *k*_1_, *k*_2_, *a*, *v* and *P*_c_. Moreover, the simulation results of different sample sizes were shown in the supplementary file, Additional file 1: Figures S1 and S2. The values of (*k*_1_, *k*_2_, *a*) were set to be (− 2, 2, 0), (− 8/3, 8/5, − 1/2), (− 8/5, 8/3, 1/2), (− 4, 4/3, − 1), and (− 4/3, 4, 1), which were intuitively presented in Fig. 2. Large absolute values of *a* meant that the U-shaped relationship was more asymmetric than that with small absolute values of *a*. Peak asymmetry factor [25] of the above (*k*_1_, *k*_2_, *a*) values were 1, 5/3, 3/5, 3, 1/3, respectively. The survival times were Weibull distributed with the decreasing (*v* = 1/2), constant (*v* = 1) and increasing (*v* = 5) hazard rates. The scale parameter of Weibull distribution was set to be 1. The censoring proportion *P*_c_ was set to be 0, 20 and 50%. For each scenario, the median method, the Q1Q3 method, the min*P* method and the optimal equal-HR method were performed to find the optimal cut-points.

### Measures of predictive performance

The predictive performance of Cox regression models fitted with covariates discretized by different methods was assessed in two aspects, which were discrimination power and overall performance (explained variance). Harrel’s concordance index (*c*-index) [26], Gönen and Heller’s concordance probability estimate (*CPE*) [27], and Graf’s integrated Brier Score (*IBS*) [28], Kent’s *R*_PM_^2^ [29] and Royston’s *R*_D_^2^ [30] were used to access the performance of Cox models. Both *c*-index and *CPE* are estimators of concordance probability. In general, the concordance probability is the probability that the one of two randomly selected patients with the shorter survival time has a higher predicted risk. Integrated Brier Score (*IBS*), which ranges from 0 to 1, measures the mean squared difference between forecast probability and true disease status. A predictive model with the lower *IBS* value has more accurate forecasts. *R*_PM_^2^ and *R*_D_^2^ both measure the proportion of the explained variance of outcomes by the model. The two-fold cross-validation approach [31] was applied to confirm the significance of the cut-points and obtain almost unbiased estimates of *HR*, *c*-index, *CPE*, *IBS*, *R*_PM_^2^ and *R*_D_^2^.

## Results

### Results of the simulation study

In this simulation study, we found that different *v* values didn’t influence the results. Therefore, we only illustrated the results when *v* was equal to 1 (constant hazards). There were similar results when (*k*_1_, *k*_2_, *a*) was set to be (− 8/3, 8/5, − 1/2) and (− 8/5, 8/3, 1/2), as well as when (*k*_1_, *k*_2_, *a*) was set to be (− 4, 4/3, − 1) and (− 4/3, 4, 1). Therefore, we only showed the results when (*k*_1_, *k*_2_, *a*) equaled (− 2, 2, 0), (− 8/5, 8/3, 1/2), and (− 4/3, 4, 1), which corresponded to symmetric, moderate asymmetric and severe asymmetric U-shaped relationships respectively.

**Fig. 3:**
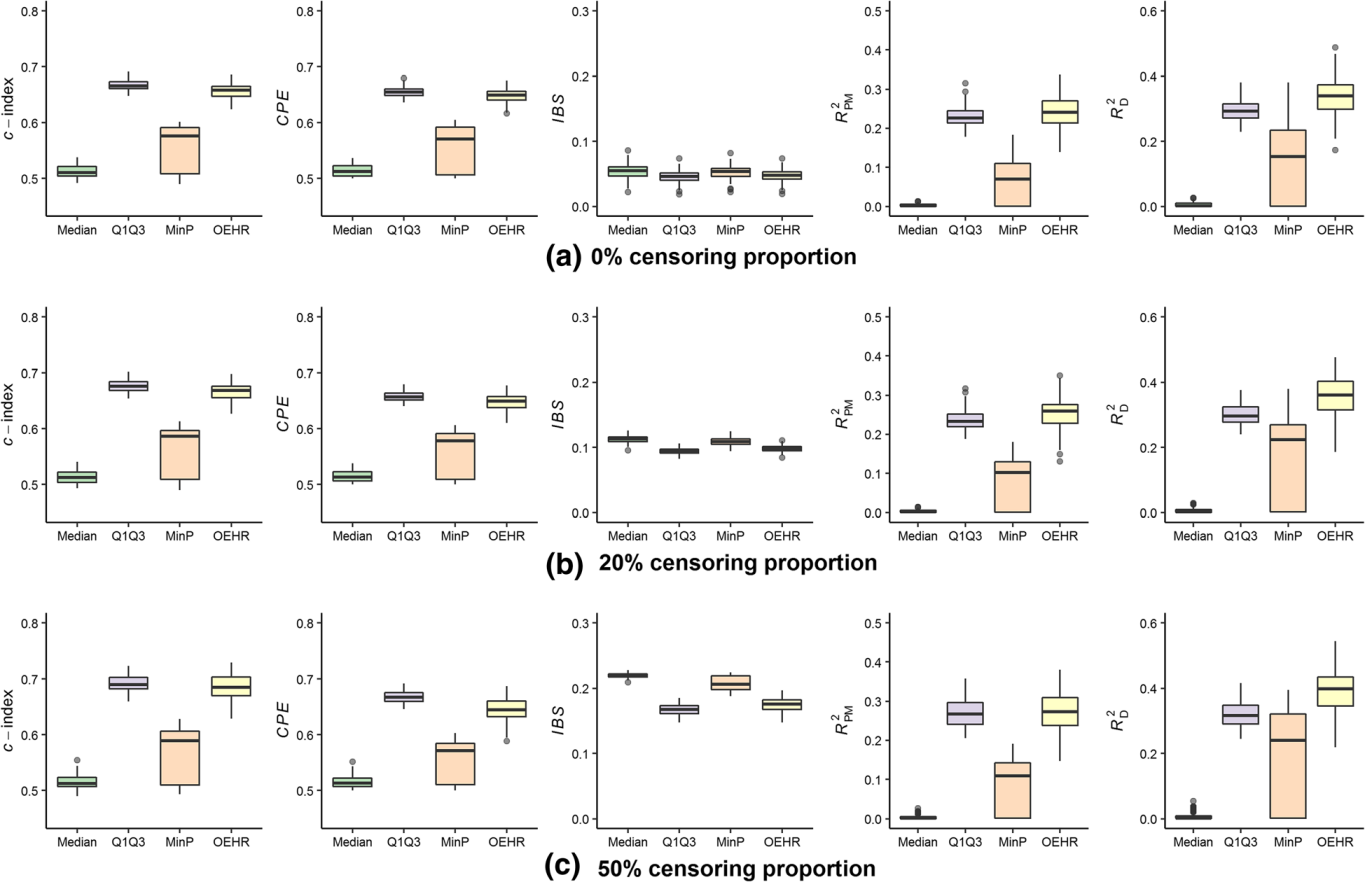
Predictive performance of estimated cut-points when (*k*_1_, *k*_2_, *a*) equals (− 2, 2, 0). **a** Simulation results when *P*_c_ = 0%; (**b**) Simulation results when *P*_c_ = 20%; (**c**) Simulation results when *P*_c_ = 50%. Four discretization methods are used to find optimal cut-points of simulated continuous variables. The continuous variables are transformed into categorical variables and then fitted in univariate Cox models. The boxplots present predictive performance of Cox models in term of *c*-index, *CPE*, *IBS*, *R*_PM_^2^ and *R*_D_^2^

**Fig. 4:**
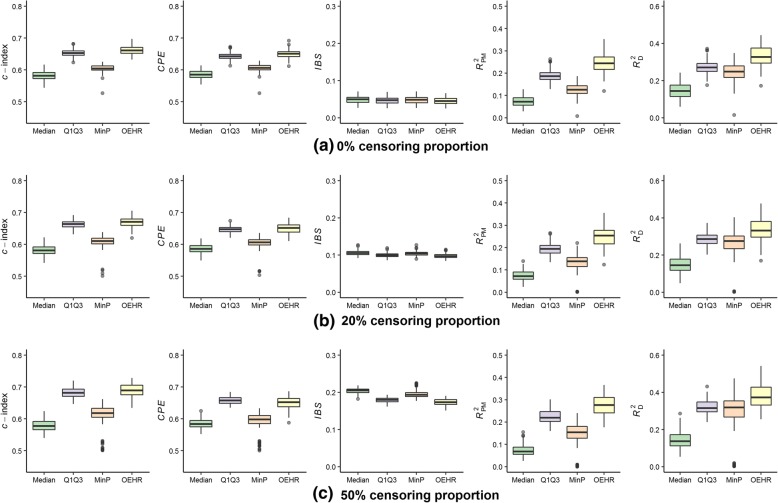
Predictive performance of estimated cut-points when (*k*_1_, *k*_2_, *a*) equals (− 8/5, 8/3, 1/2). **a** Simulation results when *P*_c_ = 0%; (**b**) Simulation results when *P*_c_ = 20%; (**c**) Simulation results when *P*_c_ = 50%. Four discretization methods are used to find optimal cut-points of simulated continuous variables. The continuous variables are transformed into categorical variables and then fitted in univariate Cox models. The boxplots present predictive performance of Cox models in term of *c*-index, *CPE*, *IBS*, *R*_PM_^2^ and *R*_D_^2^

**Fig. 5:**
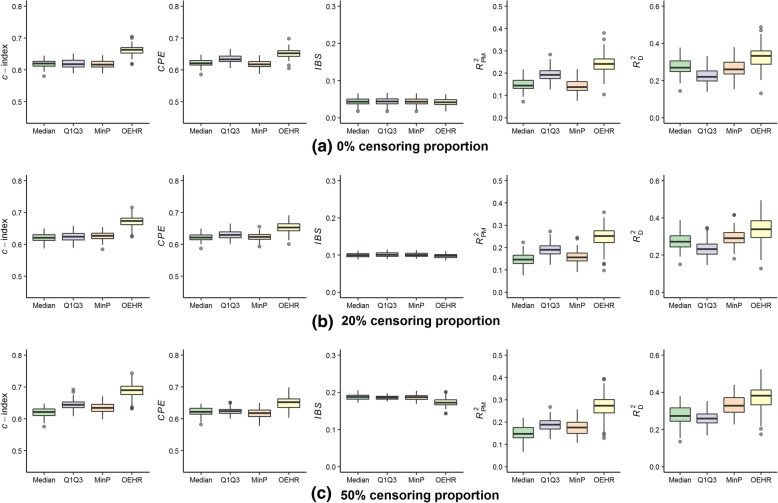
Predictive performance of estimated cut-points when (*k*_1_, *k*_2_, *a*) equals (− 4/3, 4, 1). **a** Simulation results when *P*_c_ = 0%; (**b**) Simulation results when *P*_c_ = 20%; (**c**) Simulation results when *P*_c_ = 50%. Four discretization methods are used to find optimal cut-points of simulated continuous variables. The continuous variables are transformed into categorical variables and then fitted in univariate Cox models. The boxplots present predictive performance of Cox models in term of *c*-index, *CPE*, *IBS*, *R*_PM_^2^ and *R*_D_^2^

Tables 1, 2, 3 presented the estimated cut-points of the median method, the Q1Q3 method, the min*P* method and the optimal equal-HR method under different parameter scenarios. Figures 3, 4, 5 examined the performance of Cox models with a continuous covariate discretized by the four different discretization methods.

**Table 1:** Estimated cut-points when (*k*_1_, *k*_2_, *a*) equals (− 2, 2, 0) in simulation data

Method	*P*_c_ = 0%			*P*_c_ = 20%			*P*_c_ = 50%		
	Median	Mean	Sim SE	Median	Mean	Sim SE	Median	Mean	Sim SE
Median ‘	−0.01	0.00	0.05	−0.01	0.00	0.05	−0.01	0.00	0.05
Q1Q3_1	−0.68	−0.68	0.06	−0.68	− 0.68	0.06	− 0.68	−0.68	0.06
Q1Q3_2	0.67	0.67	0.07	0.67	0.67	0.07	0.67	0.67	0.07
Min*P*	0.60	0.06	0.77	0.00	−0.02	0.84	−0.76	− 0.01	1.03
OEHR_1	−0.90	− 0.89	0.15	−0.93	− 0.93	0.16	−1.02	−1.03	0.17
OEHR_2	0.90	0.90	0.15	0.94	0.93	0.15	1.01	1.03	0.17

*P*_c_ = censoring proportion; Sim SE = simulation standard error; Median ‘= using the median value of the continuous covariate as a cut-point; Q1Q3 = using the upper and lower quartiles values as cut-points, Q1Q3_1 is the upper quartile value and Q1Q3_2 is the lower quantile value; Min*P* = the single cut-point minimum *p*-value method with log-rank test; OHER = the optimal equal-HR method proposed in this study, OEHR_1 is the left estimated cut-point and OEHR_2 is the right estimated cut-point

**Table 2:** Estimated cut-points when (*k*_1_, *k*_2_, *a*) equals (−8/5, 8/3, 1/2) in simulation data

Method	*P*_c_ = 0%			*P*_c_ = 20%			*P*_c_ = 50%		
	Median	Mean	Sim SE	Median	Mean	Sim SE	Median	Mean	Sim SE
Median ‘	−0.01	0.00	0.05	− 0.01	0.00	0.05	−0.01	0.00	0.05
Q1Q3_1	−0.68	−0.68	0.06	−0.68	− 0.68	0.06	− 0.68	−0.68	0.06
Q1Q3_2	0.67	0.67	0.07	0.67	0.67	0.07	0.67	0.67	0.07
Min*P*	−0.42	−0.40	0.13	−0.47	−0.48	0.15	−0.73	− 0.74	0.16
OEHR_1	−0.58	− 0.61	0.20	−0.65	− 0.65	0.19	− 0.80	− 0.77	0.22
OEHR_2	1.17	1.18	0.11	1.21	1.21	0.10	1.30	1.29	0.13

*P*_c_ = censoring proportion; Sim SE = simulation standard error; Median ‘= using the median value of the continuous covariate as a cut-point; Q1Q3 = using the upper and lower quartiles values as cut-points, Q1Q3_1 is the upper quartile value and Q1Q3_2 is the lower quantile value; Min*P* = the single cut-point minimum *p*-value method with log-rank test; OHER = the optimal equal-HR method proposed in this study, OEHR_1 is the left estimated cut-point and OEHR_2 is the right estimated cut-point

**Table 3:** Estimated cut-points when (*k*_1_, *k*_2_, *a*) equals (−4/3, 4, 1) in simulation data

Method	*P*_c_ = 0%			*P*_c_ = 20%			*P*_c_ = 50%		
	Median	Mean	Sim SE	Median	Mean	Sim SE	Median	Mean	Sim SE
Median ‘	−0.01	0.00	0.05	−0.01	0.00	0.05	−0.01	0.00	0.05
Q1Q3_1	−0.68	−0.68	0.06	−0.68	− 0.68	0.06	− 0.68	−0.68	0.06
Q1Q3_2	0.67	0.67	0.07	0.67	0.67	0.07	0.67	0.67	0.07
Min*P*	−0.08	−0.08	0.15	−0.19	−0.20	0.17	−0.51	− 0.51	0.20
OEHR_1	−0.41	−0.39	0.23	−0.45	− 0.46	0.24	− 0.60	−0.59	0.26
OEHR_2	1.49	1.49	0.08	1.51	1.52	0.08	1.55	1.55	0.10

*P*_c_ = censoring proportion; Sim SE = simulation standard error; Median ‘= using the median value of the continuous covariate as a cut-point; Q1Q3 = using the upper and lower quartiles values as cut-points, Q1Q3_1 is the upper quartile value and Q1Q3_2 is the lower quantile value; Min*P* = the single cut-point minimum *p*-value method with log-rank test; OHER = the optimal equal-HR method proposed in this study, OEHR_1 is the left estimated cut-point and OEHR_2 is the right estimated cut-point

When (*k*_1_, *k*_2_, *a*) equaled (− 2, 2, 0), the relationship between the continuous covariate and log(*λ*) was almost symmetric (censoring might cause mild asymmetry). The median method had the worst performance out of the four discretization methods (Fig. 3). The cut-points found by the min*P* method had much larger simulation standard errors than the other methods (Table 1). The median values of cut-points selected by the min*P* method were 0.60 (*P*_c_ = 0%), 0.00 (*P*_c_ = 20%), and − 0.76 (*P*_c_ = 50%), which also reflected the considerable variation. The min*P* method performed better than the median method, but worse than the optimal equal-HR method and the Q1Q3 method. In terms of *c*-index, *CPE,* and *IBS* measures, using Q1Q3 values as cut-points was slightly better than the other methods including the optimal equal-HR method (Fig. 3). As for *R*_PM_^2^ and *R*_D_^2^ measures, the optimal equal-HR method had the better performance than the other three methods.

When (*k*_1_, *k*_2_, *a*) equaled (− 8/5, 8/3, 1/2), the relationship between the continuous covariate and log(*λ*) was moderate asymmetric. The median value method had the worst performance among the four discretization methods (Fig. 4). In this case, the variation of cut-points found by the min*P* method was smaller than that in the symmetric situation (Table 2), and the min*P* method ranked the third regarding overall performance. The Q1Q3 method ranked the second. Overall, the optimal equal-HR method had the best performance out of the four methods. However, the advantage of the optimal equal-HR method over the Q1Q3 method was quite slight in terms of *c*-index, *CPE*, and *IBS*.

When (*k*_1_, *k*_2_, *a*) equaled (− 4/3, 4, 1), the relationship between the continuous covariate and log(*λ*) was severe asymmetric. In this case, the optimal equal-HR method had an obvious advantage over the other three methods in terms of *c*-index, *CPE, IBS*, *R*_PM_^2^ and *R*_D_^2^ measures (Fig. 5). Quantitatively, the differences between the performance of the optimal equal-HR method and the Q1Q3 method were larger than those in the moderate situation under different censoring proportions. For example, comparing the optimal equal-HR method with the Q1Q3 method, the difference of their *c*-indexes changed from 0.008 (moderate asymmetric situation) to 0.043 (severe asymmetric situation) when the *P*_c_ was 0%. Therefore, when the relationship between the continuous covariate and log(*λ*) was severe asymmetric, the optimal equal-HR method had much better performance than the Q1Q3 method, the median split method and the min*P* method.

### According to the above results, we could get the following messages

- The Q1Q3 method and the optimal equal-HR method have comparable good performance when the U-shaped relationship between the continuous covariate and log(*λ*) is almost symmetric. As the U-shaped relationship becomes asymmetric, the optimal equal-HR method has better performance than the Q1Q3 method.
- The median method and the min*P* method are not recommended when there exist U-shaped relationships between the continuous covariates and log(*λ*) since these two methods don’t have good performance under all simulation scenarios.

### Results of the application on a real dataset

Small cell lung cancer (SCLC) is a subtype of lung cancer with terrible survival outcomes. It is characterized by high invasiveness, high growth fraction, and poor prognosis. Fibrinogen (FIB), a protein that is synthesized by the liver and has a blood coagulation function, plays an important role in the pathogenesis of cardiovascular diseases. The reference range of FIB is 2–4 g/L. Several studies have found that the higher FIB level was associated with shorter overall survival (OS) [32–34]. Some authors used the median value of FIB as a cut-point of low and high FIB level [32, 33], when some others used the upper limit of the reference range as a cut-point [34].

We used the data of 275 patients with SCLC in the First Affiliated Hospital of Guangzhou Medical University from January 2009 to December 2013 [35]. FIB ranged from 0.98 to 9.23 g/L (mean ± SD: 4.78 ± 1.56 g/L). There were 235 events (deaths) during the study period. The OS ranged from 0 to 86 months (median: 12 months), and the 1-, 2-year OS rates were 50 and 21%, respectively.

**Fig. 6:**
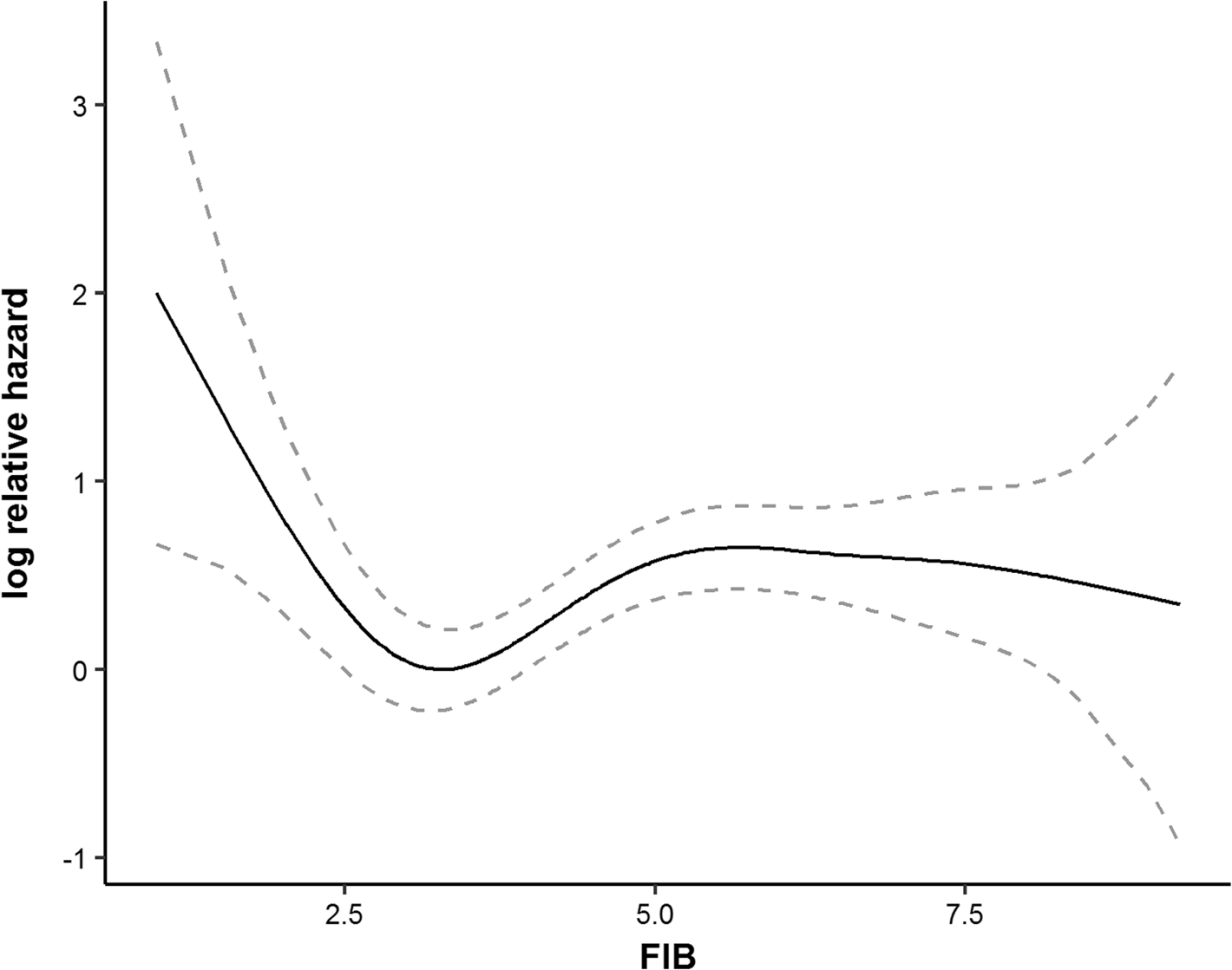
The relationship between FIB and log(*λ*) in small cell lung cancer data. The black solid line is the estimated log relative hazard of FIB by Cox model with *P*-spline, the grey dashed lines present 95% confidence interval of the estimated log relative hazard

First, the effect of FIB on log relative hazards was curved by a Cox model with *P*-splines. The U-shaped graph (Fig. 6) indicated that the patients with low and high FIB values might have a higher risk of deaths when compared to those with FIB values in the median range. It meant that the data was suitable to apply the optimal equal-HR method. Then, the median method, the Q1Q3 method, the min*P* method and the optimal equal-HR method were applied in the data.

Table 4 showed the cut-points found by the four methods. The median method selected 4.52 as a cut-point, the Q1Q3 method selected two cut-points at 3.59 and 5.84, the min*P* method selected 4.02, and the optimal equal-HR method chose 2.62 and 4.06 as cut-points. The cut-points of the optimal equal-HR method were closest to the reference range of FIB (2–4 g/L).

**Table 4:** Estimated cut-points of FIB and results of Cox models with discretized FIB

Method	*cut-point*_1_	*cut-point*_2_	*β*_1_	*HR*_1_	*P*-value_1_	*β*_2_	*HR*_2_	*p*-value_2_
Median	4.52	–	0.43	1.54	0.002	–	–	–
Q1Q3	3.59	5.84	−0.33	0.72	0.059	0.17	1.18	0.295
min*P*	4.02	–	0.46	1.59	0.001	–	–	–
OEHR	2.62	4.06	1.08	2.94	< 0.001	0.61	1.83	< 0.001

Median denotes using the median value of the continuous covariate as a cut-point. Q1Q3 denotes using the upper and lower quartiles values as cut-points. Min*P* denotes the single cut-point minimum *p*-value method with the log-rank test. OEHR denotes the optimal equal-HR method proposed in this study

Four Cox models were fitted with the discretized FIB variable. Reference levels of the discretized FIB were < 4.52 for the median method, 3.59–5.84 for the Q1Q3 method, < 4.02 for the min*P* method, and 2.62–4.06 for the optimal equal-HR method. In the Cox model with the FIB discretized by the optimal equal-HR method, both low FIB level (< 2.62) and high FIB level (> 4.06 g/L) had adverse effects (*P*-value < 0.05) on survival outcomes, while it was not statistically significant using the Q1Q3 method.

Table 5 illustrated the performance of different estimated cut-points in Cox models. 100-times five-fold cross-validation was used to obtain accurate estimations of the performance. The cut-points estimated by the optimal equal-HR method had the best performance in discrimination power and overall performance (explained variance) in terms of *c*-index, *CPE*, *IBS*, *R*_PM_^2^ and *R*_D_^2^ measures. In conclusion, the optimal equal-HR method provided clinical meaningful and well-performed cut-points in the case study.

**Table 5:** Performance of different estimated cut-points of FIB in Cox models by 100-times five-fold cross-validation

	Performance measures (mean ± standard error)				
Method	*c*-index	*CPE*	*IBS*	*R*_PM_^2^	*R*_D_^2^
Median	0.552 ± 0.006	0.554 ± 0.005	0.141 ± 0.004	0.037 ± 0.009	0.065 ± 0.017
Q1Q3	0.555 ± 0.010	0.557 ± 0.009	0.141 ± 0.005	0.040 ± 0.012	0.036 ± 0.015
min*P*	0.538 ± 0.009	0.543 ± 0.008	0.142 ± 0.004	0.027 ± 0.010	0.047 ± 0.019
OEHR	0.569 ± 0.010	0.572 ± 0.009	0.138 ± 0.006	0.069 ± 0.015	0.076 ± 0.020

Median denotes using the median value of the continuous covariate as a cut-point. Q1Q3 denotes using the upper and lower quartiles values as cut-points. Min*P* denotes the single cut-point minimum *p*-value method with the log-rank test. OEHR denotes the optimal equal-HR method proposed in this study

## Discussion

In this study, we propose the optimal equal-HR method to discretize a continuous predictor when the relationship between the predictor and log(*λ*) is U-shaped. We demonstrate the results of the Monte Carlo simulation study with different censoring proportions, baseline functions, and relationship curves. When the relationship between the predictor and log(*λ*) is symmetric (peak asymmetry factor equals 1), it’s hard to describe whether the Q1Q3 method or the optimal equal-HR method has better performance. At a first look, it surprises us a little that the Q1Q3 method has such good performance. The two possible reasons for the good performance of the Q1Q3 method are as follows. One is that under the symmetric scenario, the Q1Q3 method finds two cut-points with close log(*λ*) values, which conforms to the principle of the optimal equal-HR method. The other is that the Q1Q3 method divides samples into three groups with 1:2:1 sample sizes while the optimal equal-HR method might result in more unbalanced ratios of sample sizes. When the relationship becomes asymmetric, which is common in practice, the optimal equal-HR method has better performance than the Q1Q3 method, the median method, and the min*P* method. The SCLC case study has also proved that the cut-points selects by the optimal equal-HR method have more plausible biological sense and better model performance in the asymmetric situation.

We have only used the optimal equal-HR method under univariate Cox regression models in this study. The effect of continuous predictors on survival outcome might be influenced by other confounding variables. Therefore, it is important to include those variables in Cox models. Mazumber, et al [36] suggested that when we add a new categorical predictor to an existing multivariate model, searching the cut-points for the new variable should be done in the existing multivariate setting. The optimal equal-HR method could easily extend to multivariate Cox models after the selection of confounding variables is made depend on the ground of clinical and epidemiological understandings. As mentioned before, the optimal equal-HR method includes two main steps: (1). the graphical diagnostic plots, (2). find two optimal cut-points. The optimal equal-HR method could be applied in a multivariate context as follows. First, the diagnostic plots and estimated log relative hazards could be obtained from a multivariate Cox regression model with penalized B-splines, which means the functional form for the interested covariate is determined under the condition that all other covariates remain constant. Then, the optimal cut-points of the interested covariate will be the cut-points in the multivariate Cox regression model with minimum *AIC* value. Additionally, the simulation results in Additional file 1: Tables S4 and S5 showed that applying our method in a multivariate context will provide more reasonable estimates of both optimal cut-points (smaller variations) and Cox regression coefficients (closer to the true regression coefficients) than that in a univariate context if the survival outcomes are truly affected by other covariates. Nevertheless, the R package ‘CutpointsOEHR’ already supports seeking optimal cut-points in a multivariate context. However, when there are two or more covariates that need to be categorized, the implementation of the optimal equal-HR method remains to be addressed in further studies.

Other researchers have also proposed different ways to find two optimal cut-points. Camp, et al [37] developed X-Tile, a bio-informatics tool to find optimal cut-points, which is widely used in genetic data. The X-Tile accesses all combinations of two cut-points and selects the one with the highest log-rank *χ*^2^ value as the optimal pair of cut-points. Compared to the X-Tile, the optimal equal-HR method finds two cut-points with approximately equal log(*λ*) values, which is based on the underlying idea of classifying high-risk or low-risk population according to their log relative hazards. In this way, the optimal equal-HR method might provide more clinical valuable cut-points than the X-tile, offer clues for finding reasonable reference ranges, and reduce the number of computer operations. We might expect a better prediction performance if we allow different hazard values for two cut-points. Therefore, it is possible to improve our strategy with a trade-off between prediction performance and clinical meaning in further studies.

There are more works to be done in the future to generalize the utilization of the optimal equal-HR method: (1) This method uses Cox models with penalized B-splines to curve U-shaped relationships, which requires covariates to satisfy the proportional hazard (PH) assumption. Therefore, further studies are needed to release the PH assumption. (2) We focus on the U-shaped relationships between covariates and log relative hazards in this study. The modification and application of the optimal equal-HR method to other types of nonlinear relationships remain to be explored.

It is important to remember that lots of literature have proved that discretization will inevitably result in information loss [38–40]. This study did not encourage researchers to blindly discretize continuous independent variables when fitting Cox models. The decision to discretize a continuous covariate should be cautiously made by the investigators based on clinical needs. What’s more, the precondition of our method is the U-shaped relationship between an interested continuous covariate and survival outcomes. We recommend using the graphical diagnostic plots, which are based on Cox models with penalized *B*-splines, to visualize the data and determine whether there exists U-shaped relationships or not. Besides the graphical diagnostic plots, formal tests, such as the two-lines test [41], could also facilitate the judgement of U-shaped relationships.

## Conclusions

In general, the optimal equal-HR method proposed in our study offers researchers a solution to find optimal cut-points of continuous predictors that have U-shaped relationships with log(*λ*) in survival analysis. If researchers have decided to discretize a continuous predictor in Cox models, we highly advise them to explore the relationships between continuous predictors and survival outcomes firstly. When the relationships are U shapes, of which the majority are asymmetric in real-world data, the optimal equal-HR method is recommended to find two optimal cut-points. In addition, an R package called ‘CutpointsOEHR’ has been developed for easy use of this methodology in practice.

## Additional file

Additional file 1: **Table S1.** Comparison of the cut-points selected by the optimal equal-HR method using *AIC* and *BIC* in simulated datasets when (*k*_1_, *k*_2_, *a*) equals (− 2, 2, 0). **Table S2.** Comparison of the cut-points selected by the optimal equal-HR method using *AIC* and *BIC* in simulated datasets when (*k*_1_, *k*_2_, *a*) equals (− 8/5, 8/3, 1/2). **Table S3.** Comparison of the cut-points selected by the optimal equal-HR method using *AIC* and *BIC* in simulated datasets when (*k*_1_, *k*_2_, *a*) equals (− 4/3, 4, 1). **Table S4.** The estimated cut-points ^a^ by the multivariate and univariate approaches of the optimal equal-HR method. **Table S5.** The estimated Cox regression coefficients ^a^ of covariates discretized by the multivariate and univariate approaches of the optimal equal-HR method. **Figure S1.** Predictive performance of estimated cut-points when sample sizes are 250. **Figure S2.** Predictive performance of estimated cut-points when sample sizes range from 100 to 500 (DOCX 441 kb)

## Abbreviations

*AIC*: Akaike Information Criterion
CPE: Concordance probability estimate
HR: Hazard ratio
IBS: Integrated brier score
Min*P*: Minimum *p*-value approach to find optimal cut-points
*P*_c_: Censoring proportion
Q1Q3: Upper quantile and lower quantile values
SE: Standard error
