# Supplementary material for: A novel approach to determine two optimal cut-points of a continuous predictor with a U-shaped relationship to hazard ratio in survival data: simulation and application
Source: BMC Med Res Methodol. 2019 May 9;19:96. doi: 10.1186/s12874-019-0738-4 (PMC6507062; doi:10.1186/s12874-019-0738-4)
Supplement: Supplementary file 1 — Table S1. Comparison of the cut-points selected by the optimal equal-HR method using AIC and BIC in simulated datasets when (k1, k2, a) equals (− 2, 2, 0). Table S2. Comparison of the cut-points selected by the optimal equal-HR method using AIC and BIC in simulated datasets when (k1, k2, a) equals (− 8/5, 8/3, 1/2). Table S3. Comparison of the cut-points selected by the optimal equal-HR method using AIC and BIC in simulated datasets when (k1, k2, a) equals (− 4/3, 4, 1). Table S4. The estimated cut-points a by the multivariate and univariate approaches of the optimal equal-HR method. Table S5. The estimated Cox regression coefficients a of covariates discretized by the multivariate and univariate approaches of the optimal equal-HR method. Figure S1. Predictive performance of estimated cut-points when sample sizes are 250. Figure S2. Predictive performance of estimated cut-points when sample sizes range from 100 to 500 (DOCX 441 kb) [file 12874_2019_738_MOESM1_ESM.docx]

| Table S1. Comparison of the cut-points selected by the optimal equal-HR method using *AIC* and *BIC* in simulated datasets when (*k*_1_, *k*_2_, *a*) equals (-2, 2, 0) | | | | | | | | | | | | |  |
| --- | --- | --- | --- | --- | --- | --- | --- | --- | --- | --- | --- | --- | --- |
| *P*_c_ | Cut-points | *AIC* | | |  | *BIC* | | |  | Difference | | | |
|  |  | Median | Mean | Sim SE |  | Median | Mean | Sim SE |  | Median | Mean | Sim SE | |
| 0% | L | -0.90 | -0.90 | 0.15 |  | -0.90 | -0.90 | 0.15 |  | 0.00 | 0.00 | 0.00 | |
|  | R | 0.91 | 0.90 | 0.14 |  | 0.91 | 0.90 | 0.14 |  | 0.00 | 0.00 | 0.00 | |
| 20% | L | -0.90 | -0.90 | 0.16 |  | -0.90 | -0.90 | 0.16 |  | 0.00 | 0.00 | 0.00 | |
|  | R | 0.91 | 0.91 | 0.16 |  | 0.91 | 0.91 | 0.16 |  | 0.00 | 0.00 | 0.00 | |
| 50% | L | -1.05 | -1.05 | 0.17 |  | -1.05 | -1.05 | 0.17 |  | 0.00 | 0.00 | 0.00 | |
|  | R | 1.05 | 1.05 | 0.17 |  | 1.05 | 1.05 | 0.17 |  | 0.00 | 0.00 | 0.00 | |
| *P*c = censoring proportion; Sim SE = simulation standard error; L is the left estimated cut-point and R is the right estimated cut-point; difference= cut-point (using *AIC*) – cut-point (using *BIC*). | | | | | | | | | | | | |  |

| Table S2. Comparison of the cut-points selected by the optimal equal-HR method using *AIC* and *BIC* in simulated datasets when (*k*_1_, *k*_2_, *a*) equals (-8/5, 8/3, 1/2) | | | | | | | | | | | | |
| --- | --- | --- | --- | --- | --- | --- | --- | --- | --- | --- | --- | --- |
| *P*_c_ | Cut-points | *AIC* | | |  | *BIC* | | |  | Difference | | |
|  |  | Median | Mean | Sim SE |  | Median | Mean | Sim SE |  | Median | Mean | Sim SE |
| 0% | L | -0.61 | -0.57 | 0.20 |  | -0.61 | -0.57 | 0.20 |  | 0.00 | 0.00 | 0.00 |
|  | R | 1.18 | 1.17 | 0.11 |  | 1.18 | 1.17 | 0.11 |  | 0.00 | 0.00 | 0.00 |
| 20% | L | -0.66 | -0.65 | 0.22 |  | -0.66 | -0.65 | 0.22 |  | 0.00 | 0.00 | 0.00 |
|  | R | 1.22 | 1.22 | 0.12 |  | 1.22 | 1.22 | 0.12 |  | 0.00 | 0.00 | 0.00 |
| 50% | L | -0.77 | -0.79 | 0.21 |  | -0.77 | -0.79 | 0.21 |  | 0.00 | 0.00 | 0.00 |
|  | R | 1.30 | 1.29 | 0.14 |  | 1.30 | 1.29 | 0.14 |  | 0.00 | 0.00 | 0.00 |
| *P*c = censoring proportion; Sim SE = simulation standard error; L is the left estimated cut-point and R is the right estimated cut-point; difference= cut-point (using *AIC*) – cut-point (using *BIC*). | | | | | | | | | | | | |

| Table S3. Comparison of the cut-points selected by the optimal equal-HR method using *AIC* and *BIC* in simulated datasets when (*k*_1_, *k*_2_, *a*) equals (-4/3, 4, 1) | | | | | | | | | | | | |
| --- | --- | --- | --- | --- | --- | --- | --- | --- | --- | --- | --- | --- |
| *P*_c_ | Cut-points | *AIC* | | |  | *BIC* | | |  | Difference | | |
|  |  | Median | Mean | Sim SE |  | Median | Mean | Sim SE |  | Median | Mean | Sim SE |
| 0% | L | -0.39 | -0.40 | 0.23 |  | -0.39 | -0.40 | 0.23 |  | 0.00 | 0.00 | 0.00 |
|  | R | 1.49 | 1.49 | 0.08 |  | 1.49 | 1.49 | 0.08 |  | 0.00 | 0.00 | 0.00 |
| 20% | L | -0.43 | -0.42 | 0.25 |  | -0.43 | -0.42 | 0.25 |  | 0.00 | 0.00 | 0.00 |
|  | R | 1.51 | 1.51 | 0.09 |  | 1.51 | 1.51 | 0.09 |  | 0.00 | 0.00 | 0.00 |
| 50% | L | -0.58 | -0.54 | 0.24 |  | -0.58 | -0.54 | 0.24 |  | 0.00 | 0.00 | 0.00 |
|  | R | 1.56 | 1.55 | 0.09 |  | 1.56 | 1.55 | 0.09 |  | 0.00 | 0.00 | 0.00 |
| *P*c = censoring proportion; Sim SE = simulation standard error; L is the left estimated cut-point and R is the right estimated cut-point; difference= cut-point (using *AIC*) – cut-point (using *BIC*). | | | | | | | | | | | | |


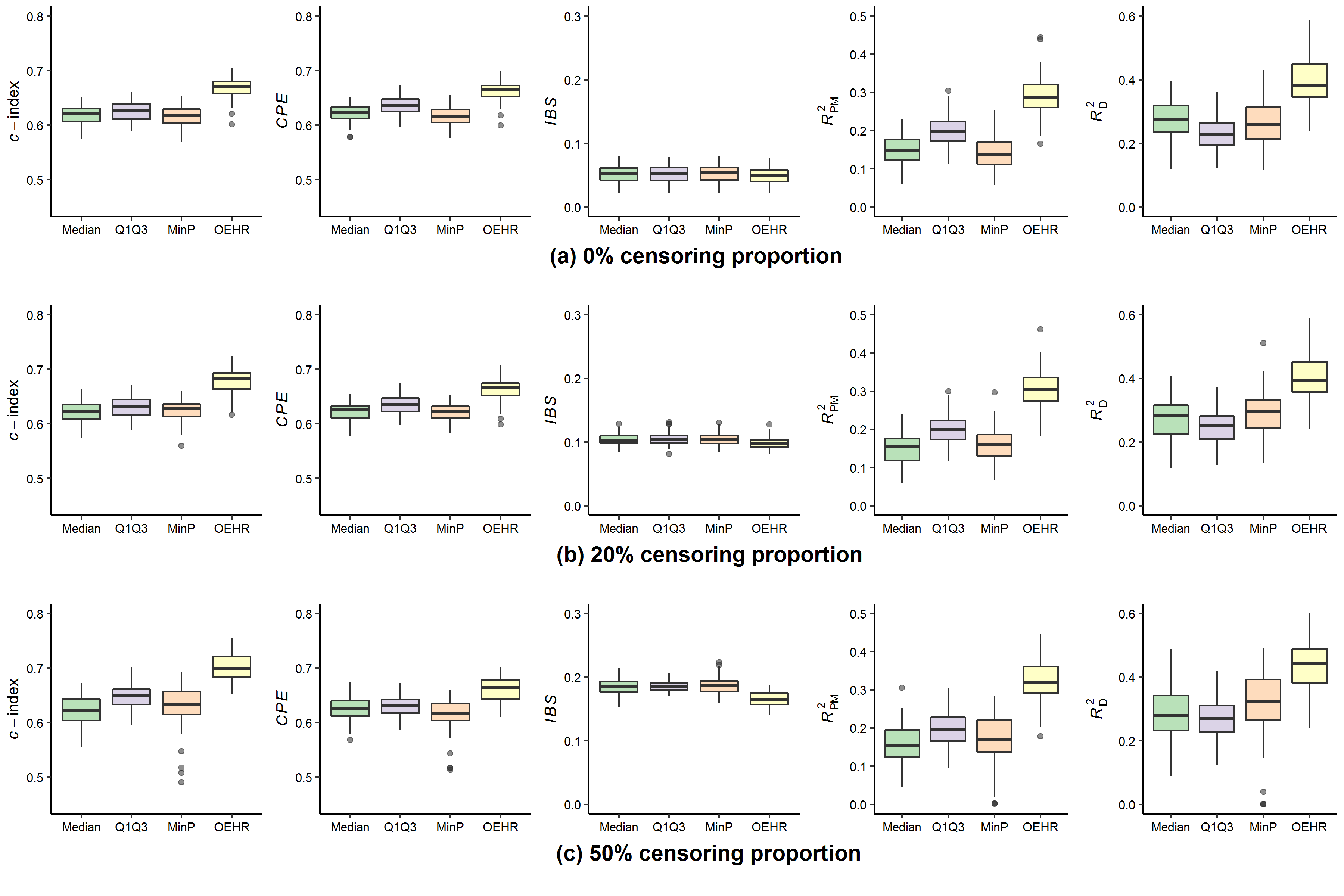


Figure S1. **Predictive performance of estimated cut-points when sample sizes are 250.** Four discretization methods are used to find optimal cut-points of continuous variables in 100 replicates of simulated data when (*k*_1_, *k*_2_, *a*) equals (-4/3, 4, 1). The continuous variables are transformed into categorical variables and then fit in univariate Cox models. The boxplots present predictive performance of the Cox models in term of *c*-index, *CPE*, *IBS*, *R*_PM_^2^ and *R*_D_^2^.


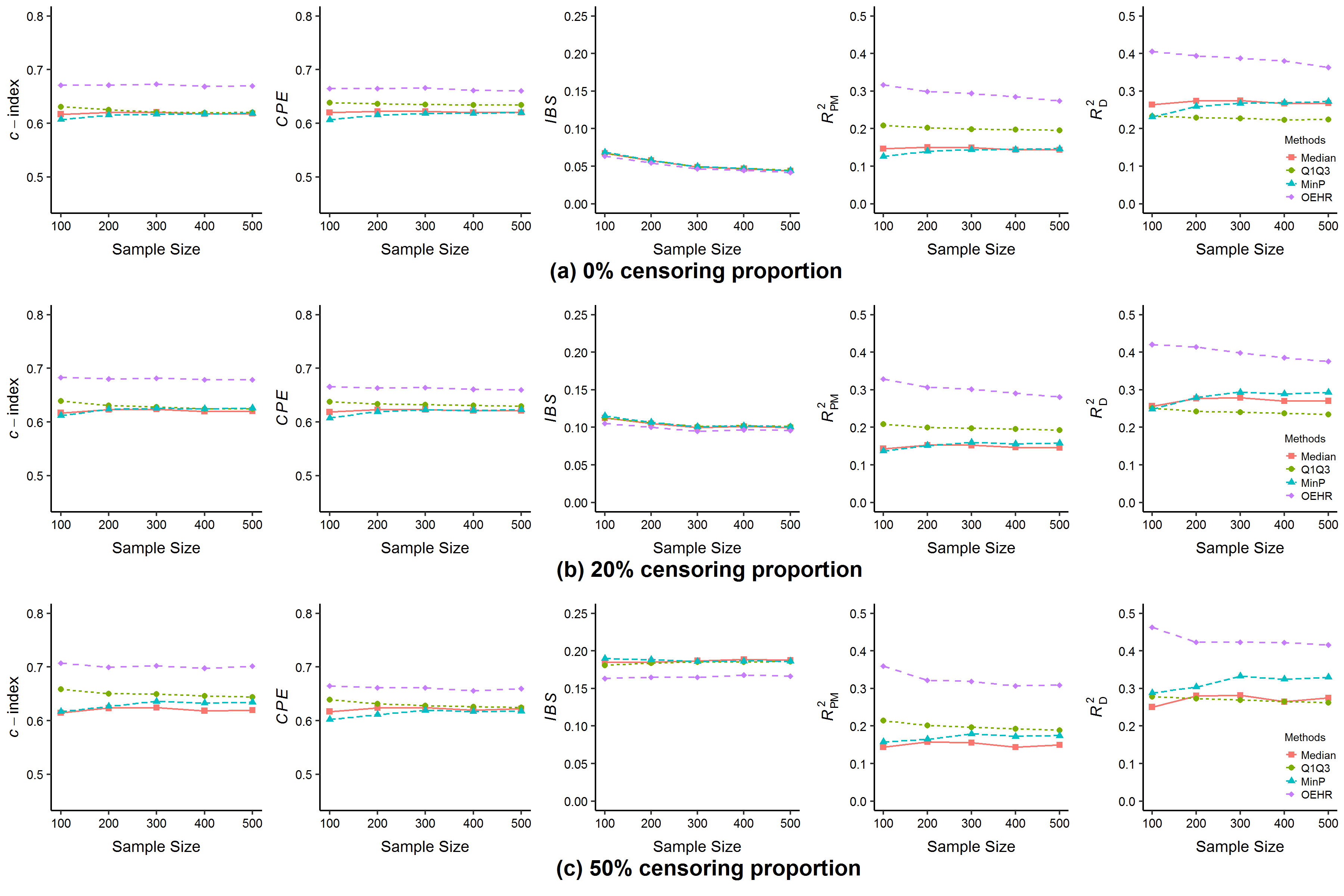


Figure S2. **Predictive performance of estimated cut-points when sample sizes range from 100 to 500.** Four discretization methods are used to find optimal cut-points of continuous variables when (*k*_1_, *k*_2_, *a*) equals (-4/3, 4, 1) in 100 replicates of simulated data. The line charts present the average predictive performance (mean values) of estimated cut-points by four discretization methods in term of *c*-index, *CPE*, *IBS*, *R*_PM_^2^ and *R*_D_^2^.

| Table S4. The estimated cut-points ^a^ by the multivariate and univariate approaches of the optimal equal-HR method. | | | | | | | | | |
| --- | --- | --- | --- | --- | --- | --- | --- | --- | --- |
|  |  | Scenario I | |  | Scenario II | |  | Scenario III | |
| *P*_c_ |  | L | R |  | L | R |  | L | R |
| 0% | MV | -0.92 ± 0.15 | 0.92 ± 0.16 |  | -0.64 ± 0.18 | 1.21 ± 0.11 |  | -0.36 ± 0.24 | 1.49 ± 0.08 |
|  | UV | -0.99 ± 0.29 | 1.04 ± 0.31 |  | -0.82 ± 0.37 | 1.34 ± 0.24 |  | -0.53 ± 0.47 | 1.54 ± 0.18 |
| 20% | MV | -0.94 ± 0.16 | 0.94 ± 0.18 |  | -0.70 ± 0.21 | 1.24 ± 0.13 |  | -0.46 ± 0.24 | 1.52 ± 0.08 |
|  | UV | -1.01 ± 0.29 | 1.05 ± 0.32 |  | -0.79 ± 0.36 | 1.33 ± 0.22 |  | -0.58 ± 0.45 | 1.58 ± 0.16 |
| 50% | MV | -1.02 ± 0.21 | 1.01 ± 0.21 |  | -0.81 ± 0.23 | 1.30 ± 0.14 |  | -0.56 ± 0.25 | 1.55 ± 0.10 |
|  | UV | -1.02 ± 0.30 | 1.04 ± 0.30 |  | -0.84 ± 0.36 | 1.36 ± 0.23 |  | -0.61 ± 0.43 | 1.60 ± 0.16 |
| ^a^ The mean and standard error of estimated cut-points over 100 simulated data (n = 500) are shown.  **Abbreviations**: *P*_c_ = censoring proportion, MV = the multivariate approach, UV = the univariate approach, L is the left estimated cut-point and R is the right estimated cut-point  **Simulation settings**: We consider scenario I, II, III with (*k*_1_, *k*_2_, a) that equals (-2, 2, 0), (-8/5, 8/3, 1/2), (-4/3, 4, 1) respectively to generate an interested covariate from a standard normal distribution with a U-shaped relationship to survival outcomes. Except the interested covariate, we generate three independent covariates from a standard normal distribution whose true Cox regression coefficients with survival outcomes are 0.5, 1.0 and 2.0 respectively. Other details of the simulation settings are at the methods section. The univariate approach only uses the interested covariate and survival outcomes to find optimal cut-points while the multivariate approach includes the other three covariates as well. | | | | | | | | | |

| Table S5. The estimated Cox regression coefficients ^a^ of covariates discretized by the multivariate and univariate approaches of the optimal equal-HR method. | | | | | | | | | |
| --- | --- | --- | --- | --- | --- | --- | --- | --- | --- |
|  | | True Regression Coefficients | | | | | | | |
|  |  | Scenario I  *-k*_1_=2.00, *k*_2_=2.00 | |  | Scenario II  *-k*_1_=1.60, *k*_2_=2.67 | |  | Scenario III  *-k*_1_=1.33, *k*_2_=4.00 | |
| *P*_c_ |  | *β*_L_ | *β*_R_ |  | *β*_L_ | *β*_R_ |  | *β*_L_ | *β*_R_ |
| 0% | MV | 1.70 ± 0.20 | 1.69 ± 0.20 |  | 1.60 ± 0.20 | 1.78 ± 0.23 |  | 1.50 ± 0.16 | 1.98 ± 0.25 |
|  | UV | 0.88 ± 0.20 | 0.85 ± 0.22 |  | 0.82 ± 0.18 | 0.94 ± 0.23 |  | 0.78 ± 0.17 | 1.07 ± 0.26 |
| 20% | MV | 1.79 ± 0.23 | 1.76 ± 0.22 |  | 1.65 ± 0.20 | 1.88 ± 0.26 |  | 1.57 ± 0.16 | 2.08 ± 0.31 |
|  | UV | 0.94 ± 0.22 | 0.91 ± 0.23 |  | 0.90 ± 0.20 | 1.02 ± 0.23 |  | 0.82 ± 0.17 | 1.08 ± 0.31 |
| 50% | MV | 1.91 ± 0.23 | 1.89 ± 0.26 |  | 1.84 ± 0.21 | 2.08 ± 0.28 |  | 1.68 ± 0.22 | 2.28 ± 0.33 |
|  | UV | 1.06 ± 0.21 | 1.04 ± 0.25 |  | 1.02 ± 0.23 | 1.14 ± 0.27 |  | 0.96 ± 0.18 | 1.32 ± 0.30 |
| ^a^ The mean and standard error of the estimated Cox regression coefficients of the discretized covariates over 100 simulated data (n = 500) are shown, jointly with the true regression coefficients of the continuous covariates.  **Abbreviations**: *P*_c_ = censoring proportion, MV = the multivariate approach, UV = the univariate approach. The interested covariate is discretized into three categories according to two optimal cut-points: the left range (smaller than the left cut-point), median range (between the left cut-point and right cut-point, reference range), and right range (larger than the right cut-points). *β*_L_ = the regression coefficient of the left range, *β*_R_ = the regression coefficient of the right range.  **Simulation settings**: The same as Table S4. The parameters *-k*_1_, *k*_2_ is the true regression coefficients of the interested continuous covariates. The parameter *-k*_1_ is used instead of *k*_1_ because the *β*_L_ is a positive number when the median range of the discretized covariate is the reference range in a Cox model. | | | | | | | | | |
